# Experimental flatwise tensile strength dataset of carbon fibre reinforced plastic sandwich panels with different core material preparations

**DOI:** 10.1016/j.dib.2019.105055

**Published:** 2019-12-31

**Authors:** Djarot Widagdo, Andi Kuswoyo, Taufiq O. Nurpratama, Bambang K. Hadi

**Affiliations:** Faculty of Mechanical and Aerospace Engineering, Institut Teknologi Bandung, Jl. Ganesha 10, Bandung 40132, Indonesia

**Keywords:** Flatwise tension, Sandwich structures, ASTM C297, Composite material qualification

## Abstract

Flatwise tension strength of sandwich structure is important for designing a sandwich construction since it provides failure mechanism and debonding strength between the skin faces and the core, as well as (i) core strength and (ii) face strength of the sandwich structures. The flatwise tension strength is affected by many factores: method of core preparation, test environment, testing speed, etc. In this paper, the ambient test temperature was 23 deg C and the humidity was 65%. The testing speed was 0.5 mm/min. Four different core preparations were investigated. ASTM C297 was used as a standard method to get the strength values. Two processes were employed to cure the adhesive during core-to-face bonding. It was found out that cleaning the core with Methyl-ethyl-ketone (MEK) and drying further in an oven gives maximum flatwise tension strength of the sandwich structures, with the value of 5.9 MPa. The data base is important for both the manufacturing and design engineers. For the manufacturing engineers, the data provides a value for process qualification, while for design engineers it gives a maximum allowable strength for designing sandwich construction for tensile loads.

**Table:** Specifications Table

Subject	Aerospace Engineering
Specific subject area	Sandwich structures, Flatwise tension, Mechanical properties, Composite materials qualification
Type of data	Table Image Chart
How data were acquired	Laboratory experiment using Universal Testing Machine and a special apparatus for flatwise tension test
Data format	Raw
Parameters for data collection	Different core preparations: 1) core material was cleaned using Methyl-Ethyl-Ketone (MEK) spray without drying process, 2) core material was cleaned using MEK spray with drying process in an oven, 3) core material was cleaned using air spray without drying process, 4) core material was cleaned using air spray with drying process in an oven. Two methods to cure the adhesive during core-to-face bonding: table press and autoclave.
Description of data collection	Specimens with special apparatus according to ASTM C297 were put into the Universal Testing Machine and loaded in tension. The maximum load and the load-displacement curve were recorded before failure. This was done for different core preparation parameters.
Data source location	Bandung, Indonesia
Data accessibility	Date are accessible in this article. The complete raw data is available in http://bit.ly/DIB-Data-Flatwise-Test

**Table:** 

**Value of the Data** • The data set can be used as a reference concerning the flatwise tensile strength of core materials used for sandwich satructures incorporating carbon/epoxy composite faces. • The manufacturing engineers will use the data for process qualification in sandwich structures, while the design engineers will use the data for designing sandwich structures in order to avoid the debonding failure between the skin and the core in sandwich construction. • The dataset allows exploration on the use of different adhesive materials and core preparations to get the maximum flatwise tensile strength of sandwich materials. • The dataset provides an insight into the debonding failure between the skin and the core in sandwich construction.

## 1 Data

The data set provided herein was the result of experimental studies conducted to determine flatwise tensile strength of sandwich structures prepared using different methods of core material preparation and the core-to-face adhesive curing methods. The type of materials for sandwich structures is given in Table 1, while the core material preparation parameters are given in Table 2. A strength-deformation curves produced during the flatwise tensile tests were given in Fig. 4, while the complete maximum flatwise tensile load and strength was given in Table 3 for configuration parameters A, B, C and D. All specimens with different parameters were able to withstand above 5 MPa, with configuration B achieved the maximum flatwise tensile strength of 5.59 MPa. Fig. 5 provides failure surface of typical specimen configuration after test. The failure mode is a combination of adhesive, cohesive and core failure according to ASTM C297 [1]. The complete pictures of failure surfaces for each specimen is available from http://bit.ly/DIB-Data-Flatwise-Test. Table 4 gives summary of the failure modes of the specimens.

**Table 1:** List of materials.

Parts	Type of materials used
Face	Hexcell W3T282-42″-F593 carbon fibre epoxy fabric prepreg.
Core	Hexcell HRP-3/16-4.0 fibre glass honeycomb core with phenolic resin
Core-to-face adhesive	Cytec FM-300 M.03 epoxy film adhesive
Aluminum-to-face adhesive	Cytec FM-73 M.06 epoxy film adhesive

**Table 2:** Core material preparation parameters and the specimens numbering system.

Spec Code	Core material preparation parameters	Spec number	
		Table press process	Autoclave process
A	Core material was cleaned using Methyl-Ethyl-Ketone (MEK) spray without drying process	AT1 – AT5	AA1 – AA5
B	Core material was cleaned using Methyl-Ethyl-Ketone (MEK) spray with drying process in an oven	BT1 – BT5	BA1 – BA5
C	Core material was cleaned using air spray without drying process	CT1 – CT5	CA1 – CA5
D	Core material was cleaned using air spray with drying process in an oven	DT1 – DT5	DA1 – DA5

**Fig. 1:**
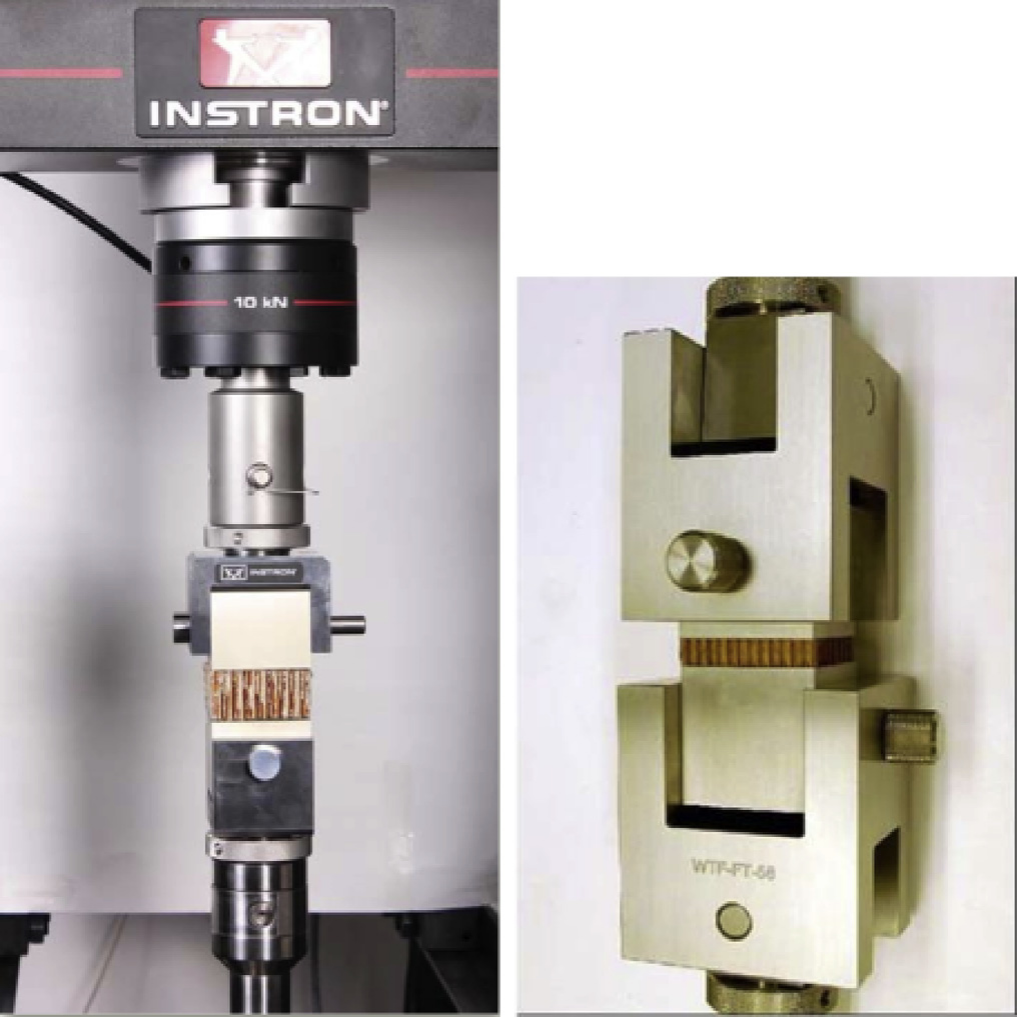
Typical flatwise tensile test ASTM C297 [1,2].

**Fig. 2:**
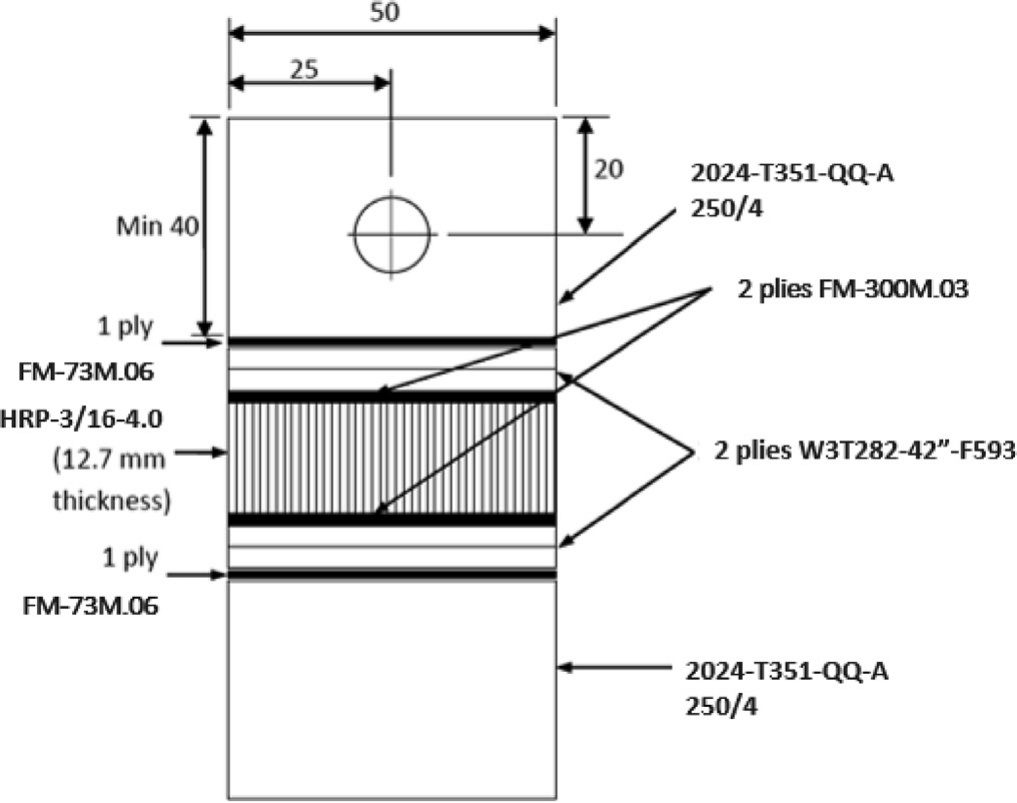
Schematic of specimens for flat-wise tensile test according to ASTM C297.

**Fig. 3:**
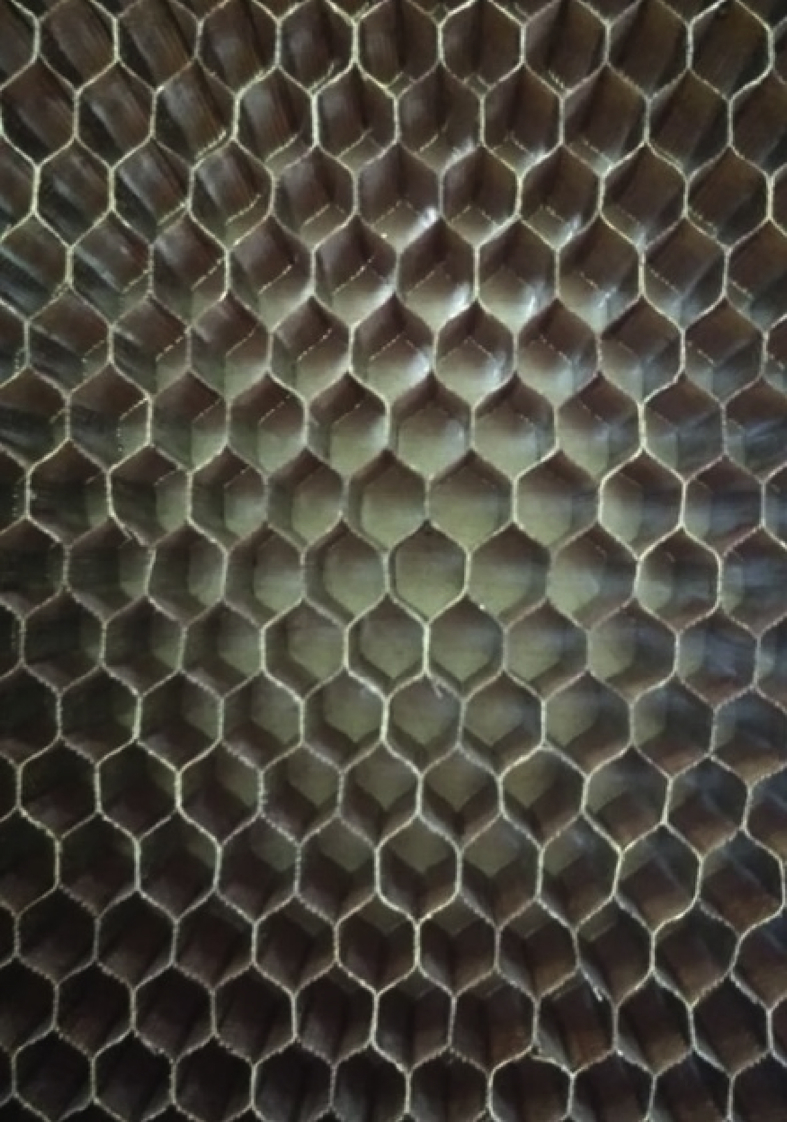
Cell core used in the experiment.

**Fig. 4:**
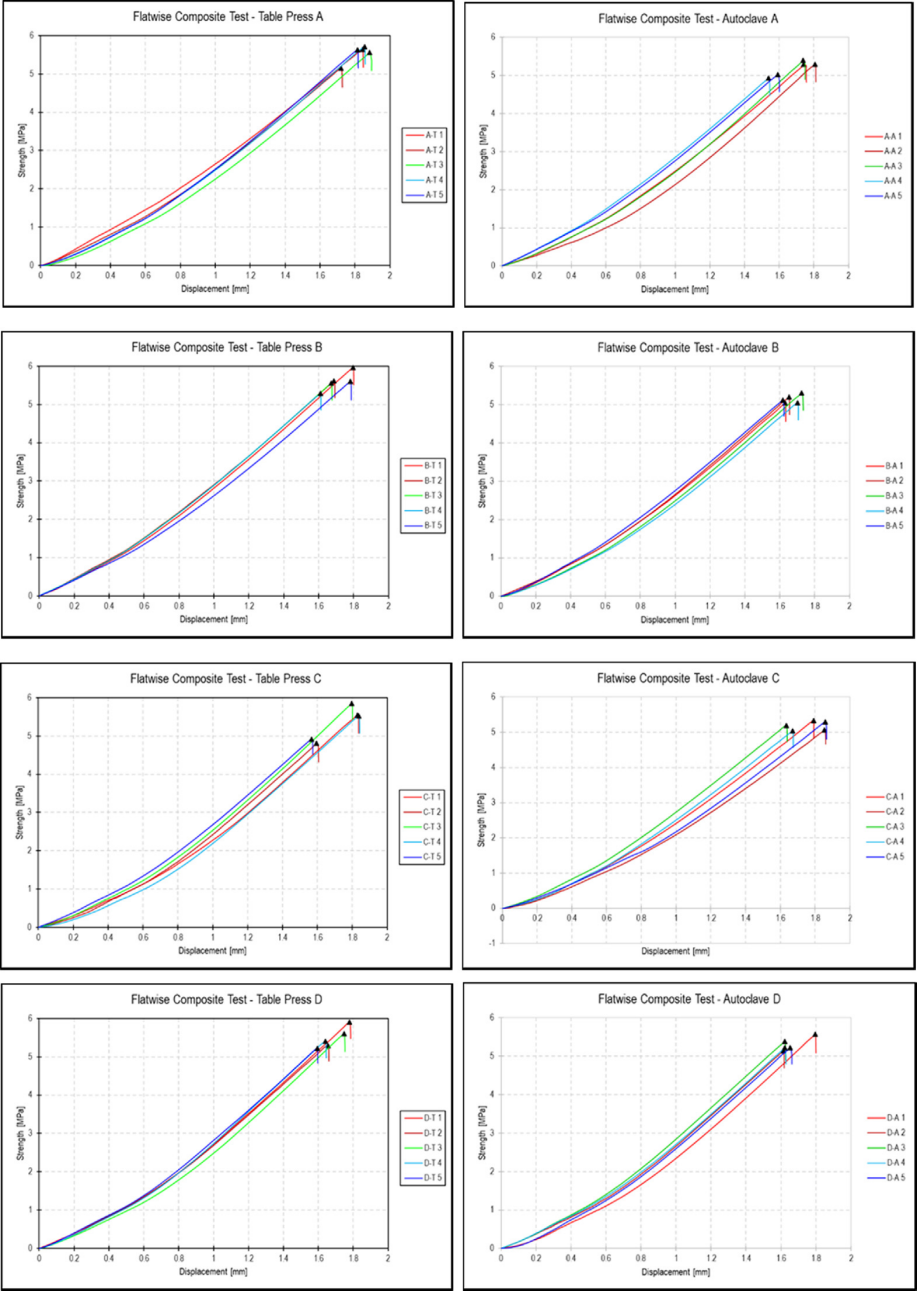
Strength-deformation results on flat-wise tensile test.

**Table 3:** Maximum load and flatwise tensile strength for different configurations.

Spec code	Table press			Autoclave		
	Spec No	Max load [N]	Flatwise strength [MPa]	Spec No	Max load [N]	Flatwise strength [MPa]
A	AT1	14354.8	5.63	AA1	13475.3	5.29
	AT2	13111.9	5.14	AA2	13480.6	5.29
	AT3	14131.6	5.54	AA3	13736.7	5.39
	AT4	14527.3	5.7	AA4	12568.4	4.93
	AT5	14312.5	5.61	AA5	12793.1	5.02
**Mean**		**14087.6**	**5.52**		**13210.8**	**5.18**
**Standard deviation**		**563.3**	**0.22**		**501.6**	**0.19**
**Coefficient of variation (%)**			**3.98**			**3.67**
B	BT1	15182.1	5.96	BA1	12841.8	5.04
	BT2	14294.0	5.61	BA2	13236.2	5.19
	BT3	14154.0	5,55	BA3	13497.2	5.29
	BT4	13450.6	5.28	BA4	12860.4	5.04
	BT5	14257.2	5.59	BA5	13047.2	5.12
**Mean**		**14267.6**	**5.59**		**13096.5**	**5.14**
**Standard deviation**		**615.9**	**0.24**		**275.2**	**0.11**
**Coefficient of variation (%)**			**4.29**			**2.14**
C	CT1	14129.7	5.54	CA1	13561.6	5.32
	CT2	12249.8	4.81	CA2	12896.7	5.06
	CT3	14880.5	5.84	CA3	13206.5	5.18
	CT4	14052.4	5.51	CA4	12815.6	5.03
	CT5	12520.4	4.91	CA5	13477.3	5.29
**Mean**		**13566.6**	**5.32**		**13191.5**	**5.18**
**Standard deviation**		**1130.7**	**0.44**		**334.3**	**0.13**
**Coefficient of variation (%)**			**8.27**			**2.51**
D	DT1	15035.2	5.90	DA1	14193.0	5.57
	DT2	13468.9	5.28	DA2	13083.1	5.13
	DT3	14255.6	5.59	DA3	13708.5	5.38
	DT4	13747.1	5.39	DA4	13304.2	5.22
	DT5	13292.6	5,21	DA5	13304.0	5.22
**Mean**		**13959.8**	**5.47**		**13518.5**	**5.30**
**Standard deviation**		**702.7**	**0.27**		**439.4**	**0.17**
**Coefficient of variation (%)**			**4.93**			**3.21**

**Fig. 5:**
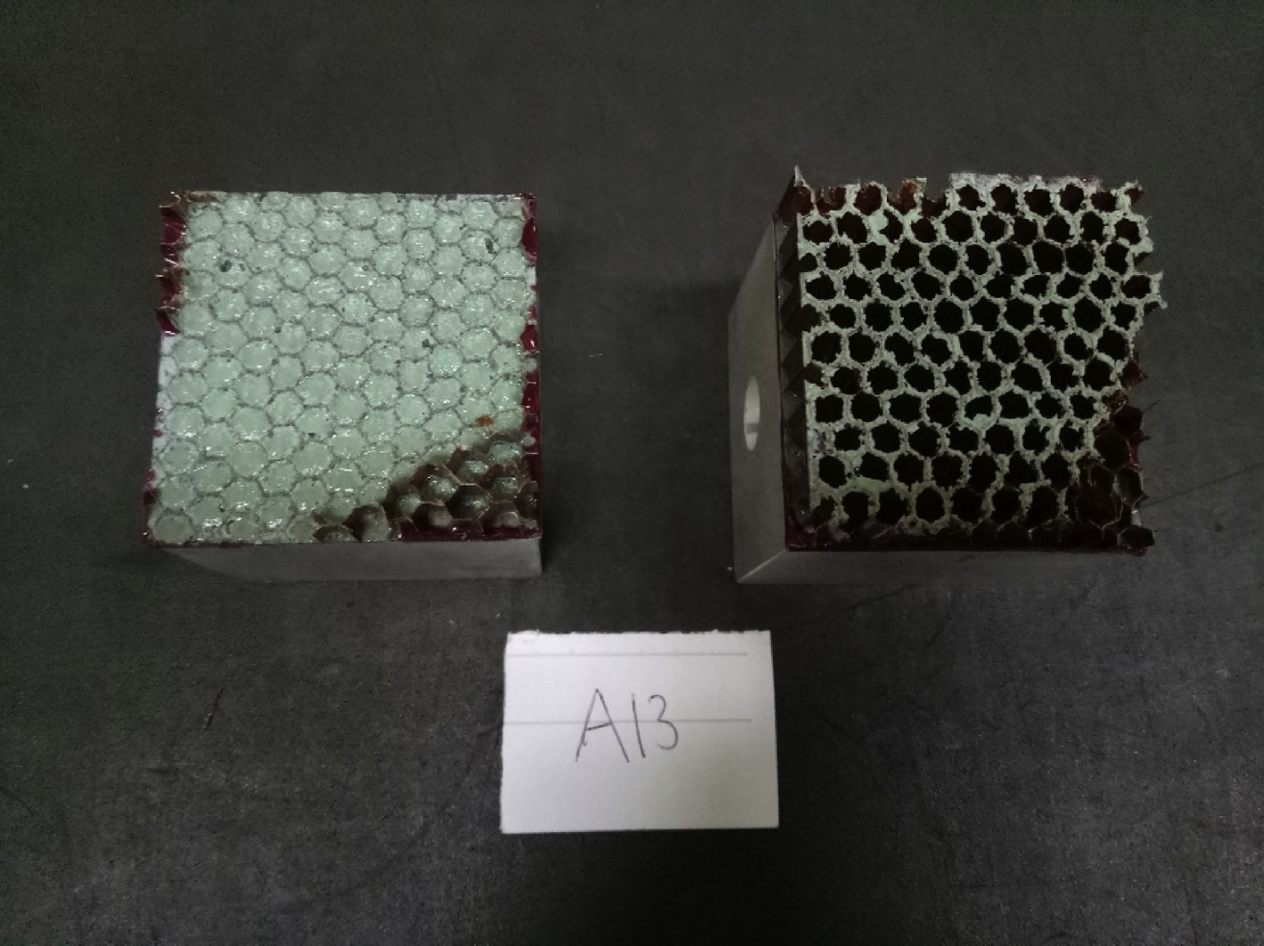
Typical failure surface of specimens after test.

**Table 4:** Summary of the failure mode of specimens according to ASTM C297.

Spec No	Failure Mode	Spec No	Failure Mode
AT1	Cohesive failure	AA1	Core failure and adhesive failure
AT2	Adhesive failure	AA2	Core failure and adhesive failure
AT3	Cohesive failure	AA3	Adhesive failure
AT4	Adhesive failure	AA4	Adhesive failure
AT5	Cohesive failure	AA5	Cohesive failure
BT1	Cohesive failure	BA1	Core failure
BT2	Core failure and adhesive failure	BA2	Adhesive failure
BT3	Core failure and cohesive failure	BA3	Cohesive failure
BT4	Core failure and adhesive failure	BA4	Core failure and cohesive failure
BT5	Core failure	BA5	Cohesive failure
CT1	Cohesive failure	CA1	Cohesive failure
CT2	Core failure and cohesive failure	CA2	Adhesive failure
CT3	Core failure and adhesive failure	CA3	Cohesive failure
CT4	Adhesive failure	CA4	Adhesive failure
CT5	Adhesive failure	CA5	Adhesive failure
DT1	Core failure and cohesive failure	DA1	Core failure and cohesive failure
DT2	Core failure and cohesive failure	DA2	Core failure and cohesive failure
DT3	Core failure and cohesive failure	DA3	Core failure and cohesive failure
DT4	Core failure	DA4	Core failure and cohesive failure
DT5	Core failure	DA5	Core failure and cohesive failure

## 2 Experimental design, materials, and methods

### 2.1 Experimental design

The experiments were carried out according to ASTM C297 [1]. Typical experimental setup is given in Fig. 1 [1,2], while the current experimental configuration is given in Fig. 2.

### 2.2 Materials

The materials used in the experimental was given in Table 1. The materials properties are as follows:

• *Core*: Fibreglass honeycomb core, HRP-3/16-4.0 Hexcell with the cell size of 3/16 inch and the density 0.064 g/cm^3^. The shear strength in L-direction is typically 4.07 MPa and the modulus 90 MPa; while in the W-direction, the strength is typically 1.10 MPa and the modulus is 45 MPa [3]. The thickness of the core was 12.7 mm.
• *Core-to-face adhesive*: FM-300 M.03 epoxy film adhesive Cytec Engineering Materials. The thickness of each ply was 0.13 mm with the density of 0.025 g/cm^3^. The service temperature was −55 deg C up to 135 deg C. The curing temperature was 170–185 deg C. Two plies of adhesive film was needed during the experiment.
• *Face:* carbon fibre fabric epoxy prepreg W3T282-42″-F593 Hexcell. The tensile strength was 3530 MPa and the modulus was 230 GPa. The thickness for each ply was 0.21 mm. Two plies of carbon/epoxy fabric was needed to produce the faces.
• *Aluminum-to-face adhesive:* FM-73 M.06, epoxy film adhesive Cytec Engineering Materials. The thickness of each ply was 0.25 mm and the density was 0.025 g/cm3. The service temperature was −55 to 80 deg C, while the curing temperature was 120–130 deg C.

#### 2.2.1 Manufacturing

The specimens manufacturing are the followings:

1) The carbon composite skin facing was manufactured using 2 plies Hexcell W3T282-42″-F593 Carbon fibre epoxy fabric prepreg and cured in an autoclave with temperature of 180 ± 5 deg C and hold for 2 hours at the pressure of 3 bars. The heating up rate was kept between 1 and 3 deg C/min, while the cooling rate was kept below 3 deg C.
2) The core material was Hexcell HRP-3/16-4.0 Fibre glass honeycomb core with phenolic resin. Fig. 3 shows core material used in the experiments. The core material was prepared using different parameters as given in Table 2. The cleaning process with MEK was carried for 1 minute. The cleaning process with air gun was also done in 1 minute. The drying process was done using oven for 2 hours with temperature of 100 deg C.
3) The skin face and the core were bonded together using adhesive film Cytec FM-300 M.03 Epoxy film adhesive and cured. The curing process used two methods: using hot table press and autoclave. The curing was done at the temperature of 120 deg C. In the table press, the specimens was vacuumed to 200–250 mmHg (0.266–0.333 bar); with a pressure of 1.70 bar for 2 hours. The same parameters were done in the autoclave process.
4) The resulted sandwich structures are then bonded to the aluminium block using Cytec FM-73 M.06 Epoxy film adhesive and cured once again in table press at the temperature of 120 deg C for 2 hours. The aluminum block was sandblasted before bonded was carried out to the faces. The resulting sandwich structures were ready for flatwise tension tests.

### 2.3 Flatwise tensile test methods

The specimen was put into the Universal Testing Machine and given the tensile load. Fig. 1 shows typical flatwise tensile experimental setup. The specimens dimension was 50 × 50 mm and the overall thickness is 94 mm. The tensile machine was Instron with the maximum capacity of 100 kN. The displacement rate was set to be in the order of 0.5 mm/minute. Fig. 1 shows the experimental setup. The maximum load and the load-displacement curve were obtained after the test.

The ultimate flatwise tensile strength was determined using the Eq. (1):

(1) $$F_{z}^{ftu}=\frac{P_{max}}{A}$$

where:

$F_{z}^{ftu}$: flatwise tensile strength

$P_{max}$: ultimate tensile load

A: area of the specimen. In this case 50 × 50 mm.

It was known that the flatwise tensile strength was affected by the environment [4] and discolouration occured when using Cytec FM-200 adhesive [5]. The environment affect the bondline strength of core-to-face adhesive. Cocured sandwich structure provided more consistent data than precured [6]. In this paper, the tests were done with the room temperature of 23 deg C and the humidity was 65%. The faces were precured before adhesively bonded to the core.
